# Tidal Volume Single Breath Washout of Two Tracer Gases - A Practical and Promising Lung Function Test

**DOI:** 10.1371/journal.pone.0017588

**Published:** 2011-03-10

**Authors:** Florian Singer, Georgette Stern, Cindy Thamrin, Oliver Fuchs, Thomas Riedel, Per Gustafsson, Urs Frey, Philipp Latzin

**Affiliations:** 1 Division of Respiratory Medicine, Department of Paediatrics, University Children's Hospital of Bern, Bern, Switzerland; 2 Paediatric and Neonatal Intensive Care Unit, Department of Paediatrics, University Children's Hospital of Bern, Bern, Switzerland; 3 Department of Paediatrics, Central Hospital, Skövde, Sweden; 4 Department of Paediatrics, University Children's Hospital Basel, Basel, Switzerland; Ludwig-Maximilians-Universität München, Germany

## Abstract

**Background:**

Small airway disease frequently occurs in chronic lung diseases and may cause ventilation inhomogeneity (VI), which can be assessed by washout tests of inert tracer gas. Using two tracer gases with unequal molar mass (MM) and diffusivity increases specificity for VI in different lung zones. Currently washout tests are underutilised due to the time and effort required for measurements. The aim of this study was to develop and validate a simple technique for a new tidal single breath washout test (SBW) of sulfur hexafluoride (SF_6_) and helium (He) using an ultrasonic flowmeter (USFM).

**Methods:**

The tracer gas mixture contained 5% SF_6_ and 26.3% He, had similar total MM as air, and was applied for a single tidal breath in 13 healthy adults. The USFM measured MM, which was then plotted against expired volume. USFM and mass spectrometer signals were compared in six subjects performing three SBW. Repeatability and reproducibility of SBW, i.e., area under the MM curve (AUC), were determined in seven subjects performing three SBW 24 hours apart.

**Results:**

USFM reliably measured MM during all SBW tests (n = 60). MM from USFM reflected SF_6_ and He washout patterns measured by mass spectrometer. USFM signals were highly associated with mass spectrometer signals, e.g., for MM, linear regression r-squared was 0.98. Intra-subject coefficient of variation of AUC was 6.8%, and coefficient of repeatability was 11.8%.

**Conclusion:**

The USFM accurately measured relative changes in SF_6_ and He washout. SBW tests were repeatable and reproducible in healthy adults. We have developed a fast, reliable, and straightforward USFM based SBW method, which provides valid information on SF_6_ and He washout patterns during tidal breathing.

## Introduction

Small airway disease frequently occurs in chronic obstructive pulmonary disease, asthma, and cystic fibrosis (CF) [1]. Despite lack of respiratory symptoms in *e.g.* adult smokers [2], children with asthma [3] or CF [4]; [5], small airway malfunction may be present and is regarded as important sign of early lung disease.

Conventional lung function techniques such as spirometry are considered to be not sensitive enough to detect small airway malfunction [3]; [6]–[8]. Inert tracer gas washout tests over single or multiple breaths (SBW or MBW) provide a more sensitive alternative to spirometry in tracking small airway malfunction, *e.g.* altered ventilation inhomogeneity (VI), and they are more accurate in reflecting structural changes in lung periphery [7]; [9]–[11]. However, these tests are not commonly used in clinical routine as they rely on custom made, expensive, and bulky setups, *e.g.* mass spectrometer (MS), and require coordination of vital capacity manoeuvres for SBW or cooperation during 20 minutes of tidal breathing for MBW [12].

Recently, the ultrasonic flowmeter (USFM) technique was applied in MBW studies [13]–[16]. The USFM measures total molar mass (MM), a sum signal derived from measured gas density [15]–[18]. Tracer gas mixtures had different MM compared to air and contained a single tracer gas, either sulfur hexafluoride (SF_6_) or helium (He) [13]–[16]. MM of SF_6_ (146 g/mol) is much higher than MM of He (4 g/mol), thus SF_6_ and He distribute unequally in lung periphery where diffusion predominates. The diffusion front for He is thought to arise in the zone of the entrance to the *acinus*. In contrast, the diffusion front for SF_6_ is predicted to occur more distally within the *acinus* [19]. Different structural asymmetries within the lung zones where the diffusion fronts of He and SF_6_ arise thus lead to unequal washout patterns of He and SF_6_. Using both gases for a SBW may provide more specific information about ventilation in these peripheral lung zones [20]–[22]. A tracer gas mixture containing SF_6_ and He and exhibiting similar MM as air could be measured by an USFM to assess washout patterns of SF_6_ and He. Using the USFM for a modified SBW procedure would eliminate some shortcomings of current tracer gas washout tests in clinical routine.

The aim of this study was to develop and validate a simple technique for a new tidal SBW of SF_6_ and He using an USFM.

## Materials and Methods

### Ethics statement

The study was approved by both the Ethics Committee of the Canton of Bern, Switzerland (Kantonale Ethikkommission Bern) and the Research Ethics Committee of University Hospital Bern (Inselspital). All participants provided written informed consent for this study.

### Study design

In this feasibility study, a SBW of a double tracer gas mixture (DTG-SBW) using an USFM was applied in 13 healthy adults during tidal breathing. The subjects' mean (SD) age was 35.2 (9.4) years. Accuracy of the USFM compared to mass spectrometry (MS), and repeatability and reproducibility of this DTG-SBW test were assessed.

### Double tracer gas

SF_6_ and He, two inert tracer gases, were employed in a SF_6_/He ratio of 1/5.26 to establish similar MM of DTG compared to dry medical-grade air (28.9 g/mol). DTG contained 5% SF_6_, 26.3% He, 21% oxygen (O_2_), and balance nitrogen (N_2_) (Carbagas, Domdidier, Switzerland) and was applied in all DTG-SBW tests (n = 60).

### Tidal single breath washout

**Figure 1:**
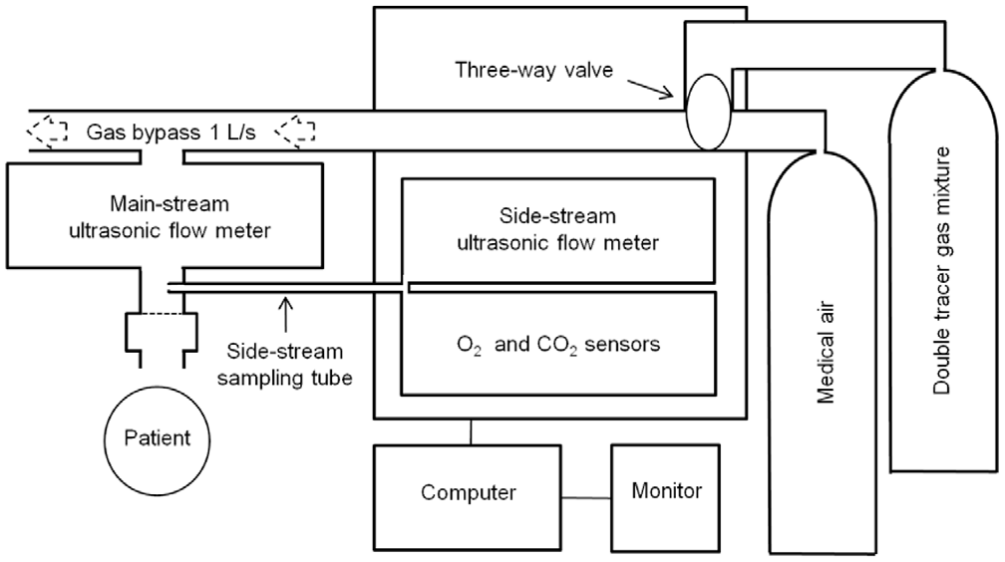
Ultrasonic flowmeter setup. Molar mass was measured in a sidestream ultrasonic flowmeter (USFM) and flow was measured in a mainstream USFM. Oxygen (O_2_), and carbon dioxide (CO_2_) were measured in sidestream sensors.

Subjects were measured in an upright sitting position, wearing a nose clip, and breathing through a disposable bacterial filter (air™ Vickers Industrial Estate, Lancashire, UK) attached to the flow-head (figure 1). Prior to the DTG-SBW, subjects tidally breathed air for 20 seconds until steady shapes of flow-volume-loops were established. At the beginning of an expiration, DTG was switched on manually to flush the system. A tidal volume of DTG was inhaled from functional residual capacity (FRC) prior to exhaling back to FRC. DTG-SBW was technically accepted if the test breath had a similar flow-volume-loop as pre-test breaths. A minimum of ten subsequent breaths of air were required prior to the next DTG-SBW, and three DTG-SBW tests were done per test occasion.

### Washout analysis

The wave form of naturally exhaled MM is attributed to the increasing carbon dioxide (CO_2_) fraction [23]. During DTG-SBW, CO_2_, SF_6_, and He fractions were expected to give rise to the USFM derived MM (MM_USFM_) signal. Therefore, we transformed the CO_2_ signal into MM and subtracted this signal from MM_USFM_. We then obtained a single MM signal potentially reflecting SF_6_ and He washout (SF_6_-He_USFM_). The main outcome of the DTG-SBW analysis was the shape of the SF_6_-He_USFM_ signal plotted as expirogram, *i.e.* MM against expired volume.

Despite some limitations, MS is still regarded as current gold standard for quantification of respiratory gases [16]; [17]. During DTG-SBW we compared MM_USFM_ and SF_6_-He_USFM_ signals with the two corresponding MS signals (MM_MS_, SF_6_-He_MS_) which were derived as follows. The MM signal (MM_MS_) was calculated by summing fractional MM of respective gas concentrations. The SF_6_ and He signals were normalized by dividing them by their starting concentrations [24]. The normalized He signal was then subtracted from the normalized SF_6_ signal to obtain a single tracer gas signal (SF_6_-He_MS_).

### Accuracy and reproducibility of the single breath washout method

Three DTG-SBW were applied in six healthy adults on a single test occasion to compare USFM signals with MS signals. MM_USFM_ and SF_6_-He_USFM_ were compared graphically with MM_MS_ and SF_6_-He_MS_. Signal-to-noise ratio of MM_USFM_ (MM_USFM_ mean/MM_USFM_ standard deviation (SD)) was assessed for air and DTG at 1 L/s flow during ten seconds.

**Figure 2:**
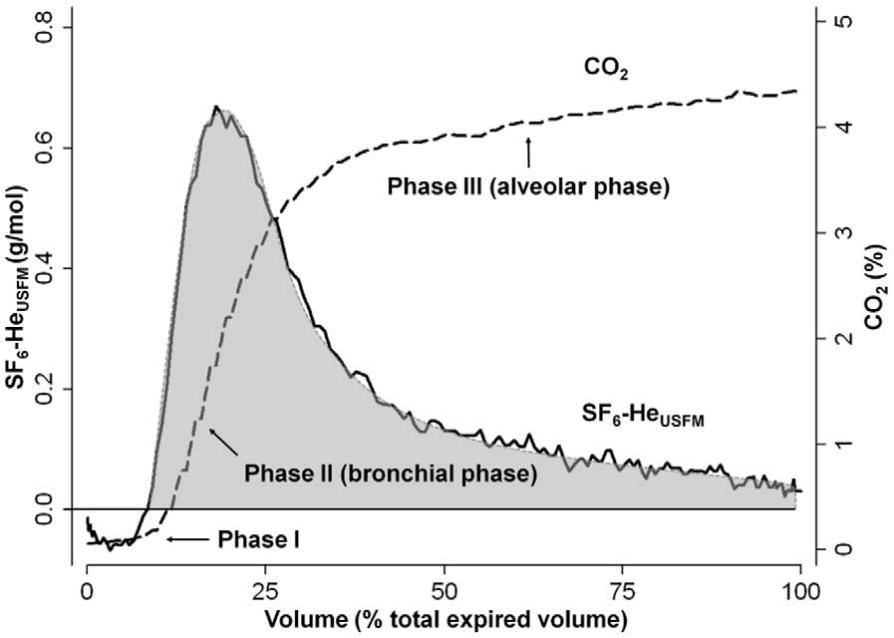
Area under the single breath washout curve. The ultrasonic flowmeter (USFM) derived sulfur hexafluoride (SF_6_) and helium (He) molar mass signal (SF_6_-He_USFM_) was plotted as expirogram. The carbon dioxide (CO_2_) expirogram (dashed line) was plotted to determine area under the washout curve (AUC, grey area) during washout of bronchial and alveolar gas fronts [25]. As SF_6_-He_USFM_ had been calculated by subtracting CO_2_ from molar mass (MM_USFM_), a “negative” SF_6_-He_USFM_ signal resulted from a low MM_USFM_ signal reflecting relatively more He than SF_6_ contribution to MM_USFM_. In phase I, relatively more He than SF_6_, but during phases II and III, relatively more SF_6_ than He sequentially arrived at the mouth.

To assess repeatability and reproducibility of the DTG-SBW test, three DTG-SBW tests were applied in seven healthy adults on two test occasions 24 hours apart. We calculated area under the washout curve (AUC) by integrating a best-fit double exponential curve (Matlab; The Mathworks Inc., Natick, MA, USA) to mathematically describe the shape of the SF_6_-He_USFM_ expirogram. We constrained AUC calculation to phases II and III of the CO_2_ expirogram (figure 2) to assess washout patterns from bronchial and alveolar gases. While during the first phase CO_2_-free gas is exhaled from airway dead space, the rapidly increasing CO_2_ fraction forms a sigmoidal curve of the bronchial phase II ending up in the plateau of the alveolar phase III [25].

### Hardware

We used a commercially available USFM setup (Exhalyzer D®, Eco Medics AG, Duernten, Switzerland) described previously (figure 1) [14]; [16]. The USFM measured MM in sidestream sampling mode with a sample flow of 200 mL/min via a Nafion® tube to allow equilibration of ambient temperature and humidity [17]; [18]. Measurement precision was 0.01 g/mol at a sampling frequency of 200 Hz [14]; [18]. Tidal flows and derived volumes were measured in mainstream gas using the flow-head USFM. Into this flow-head, a dead-space reducer (DSR size 3) and a disposable hygienic insert (Spirette), both provided by the manufacturer (Eco Medics AG), were inserted. Total dead space of the flow-head plus the bacterial filter attached was 40 mL. A three-way valve system operated manually administered air or DTG via by-pass flow at 1 L/s effecting a resistance of 0.01 kPa s L-1. Prior to measurements, the USFM was calibrated for inspiratory and expiratory volumes using a precision syringe.

Gas concentrations (SF_6_, He, N_2_, O_2_, and CO_2_) using a respiratory mass spectrometer (AMIS 2000, Innovision A/S, Odense, Denmark) and respiratory flows using a heated pneumotachograph were measured near airway opening as previously described [26]. An additional sidestream sampling Nafion® tube was introduced between the mouthpiece and the flow-head. The sample flow of the MS was 20 mL/min and the gas signals were updated at a rate of 33.3 Hz. The PC based data acquisition setup recorded flow and dry gas concentrations at 100 Hz.

### Software

A software package (WBreath® 3.28; ndd Medical Technologies, Switzerland) was used for collection of USFM and MS signals. Signals were aligned in time as previously described [16]; [26]. Tidal flows and derived volumes were converted to body temperature and ambient pressure, and saturated with water vapour (BTPS) conditions.

### Statistical analysis

The association of MM_USFM_ and SF_6_-He_USFM_ with MM_MS_ and SF_6_-He_MS_ signals were assessed graphically and using a linear regression model accounting for clustered data within individuals. Means of MM_USFM_ and MM_MS_ were compared using two-tailed paired *t*-tests. Accuracy of MM_USFM_ compared to MM_MS_ was determined using the *Bland and Altman* method [27] by plotting differences of paired measurements against means of paired measurements.

Intra-test repeatability of DTG-SBW was calculated as intra-subject mean coefficient of variation (CV%  =  SD/mean*100) of AUC. DTG-SBW curves and AUC from test occasions 24 hours apart were compared graphically, and using the two-tailed paired *t*-test. Between-test reproducibility of DTG-SBW was assessed graphically, and by the coefficient of repeatability of AUC representing the 95% range of differences between two repeated measurements and was calculated as twice the square root of the mean of squared differences of paired measurements [27]; [28]. Means, SD, and 95% confidence intervals (95% CI) were reported, p-values <0.05 were considered significant, and all analysis were done using Stata™ (StataCorp. 2009. Stata Statistical Software: Release 11. College Station, TX: StataCorp LP).

## Results

**Figure 3:**
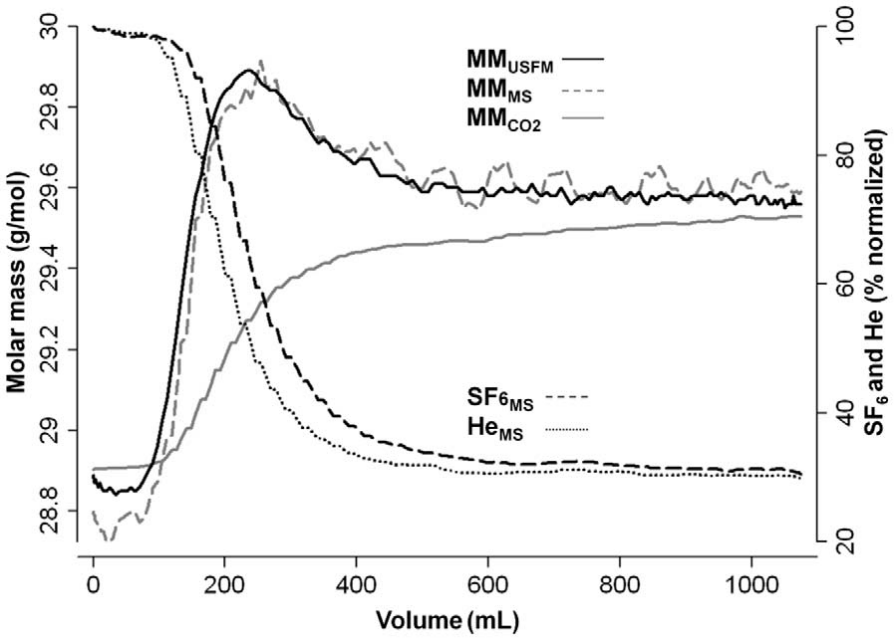
Comparison of single breath washout signals. Typical ultrasonic flowmeter (USFM) and mass spectrometer (MS) signals from a single breath washout (SBW) using sulfur hexafluoride (SF_6_) and helium (He) in a healthy male adult. USFM derived molar mass (MM_USFM_ black solid line) and MS derived molar mass (MM_MS_ grey dashed line) reflected changes in SF_6_ washout relative to He washout measured using MS: SF6_MS_ (black dashed line), and He_MS_ (black dotted line). The MM signal derived from CO_2_ (MM_CO2_ grey solid line) reflected naturally exhaled MM similar to MM signals from pre-test breaths.

DTG-SBW was feasible and technically acceptable in all subjects (n = 13; 7 males) during all tests (n = 60). Mean (SD) duration of one DTG-SBW test was 76 [27] seconds. The MM_USFM_ signal of the DTG-SBW was significantly different to the naturally exhaled MM_USFM_ signal due to CO_2_ as shown in one male subject (figure 3). Different patterns of SF_6_ and He washout was observed throughout all DTG-SBW tests reflecting a non-linear washout relationship of these tracer gases. An increase in MM_USFM_ indicated an increase in SF_6_ washout relative to He washout. This pattern was mainly observed in phase II of the CO_2_ expirogram (figures 2 and 3).

### Accuracy of the single breath washout method

**Figure 4:**
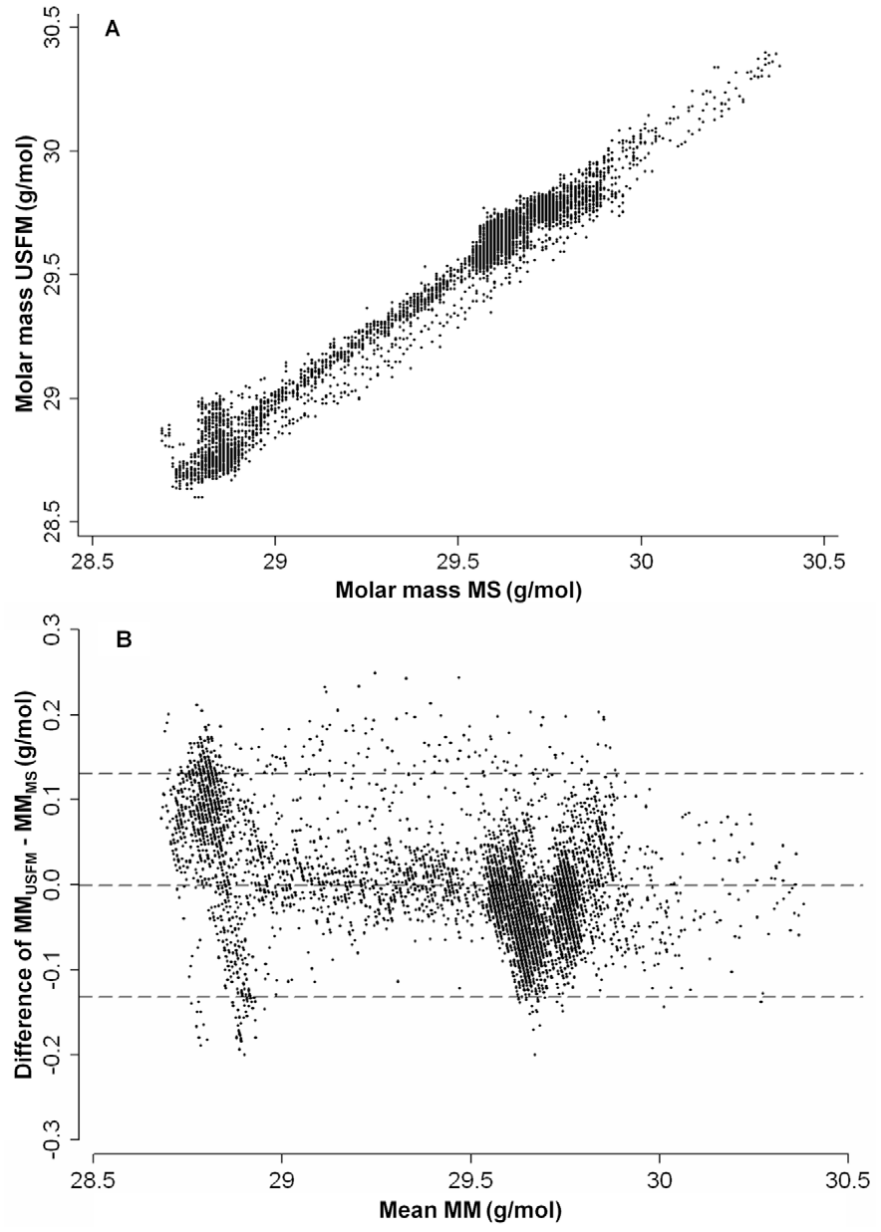
Accuracy of the ultrasonic flowmeter signal. Six healthy adults performed three tidal single breath washout tests of sulfur hexafluoride (SF_6_) and helium (He) during tidal breathing. Molar mass was measured using an ultrasonic flowmeter (MM_USFM_) and mass spectrometer (MM_MS_). Figure 4a: Paired MM_USFM_ and MM_MS_ data from 18 tidal single breath washout tests in six subjects were plotted against each other. Accounting for clustered data, adjusted linear regression coefficient r^2^ = 0.98 (p<0.001). Figure 4b: *Bland and Altman* plot of MM_USFM_ - MM_MS_ differences against mean MM of both methods [27]. Dashed lines indicated the mean difference of MM (−0.0004 g/mol), and upper and lower limits of agreement (mean of difference ± 2 SD of differences): 0.131 to −0.132 g/mol).

MM_USFM_ and MM_MS_ signals were compared using paired data from 18 tests. The association of MM_USFM_ and MM_MS_ signals was high, the adjusted linear regression coefficient r^2^ was 0.98 (figure 4a). Strong agreement of MM_USFM_ and MM_MS_ was found in the *Bland and Altman* plot (figure 4b) without graphical evidence of systematic bias or significant outliers. Mean (95% CI) difference in MM was −0.0004 (−0.0021 to 0.0013) g/mol. The range of 95% of all differences was 0.13 g/mol (0.45% of mean MM of both methods). The MM_USFM_ signal-to-noise ratio for air and DTG was 3490 and 3140, respectively.

**Figure 5:**
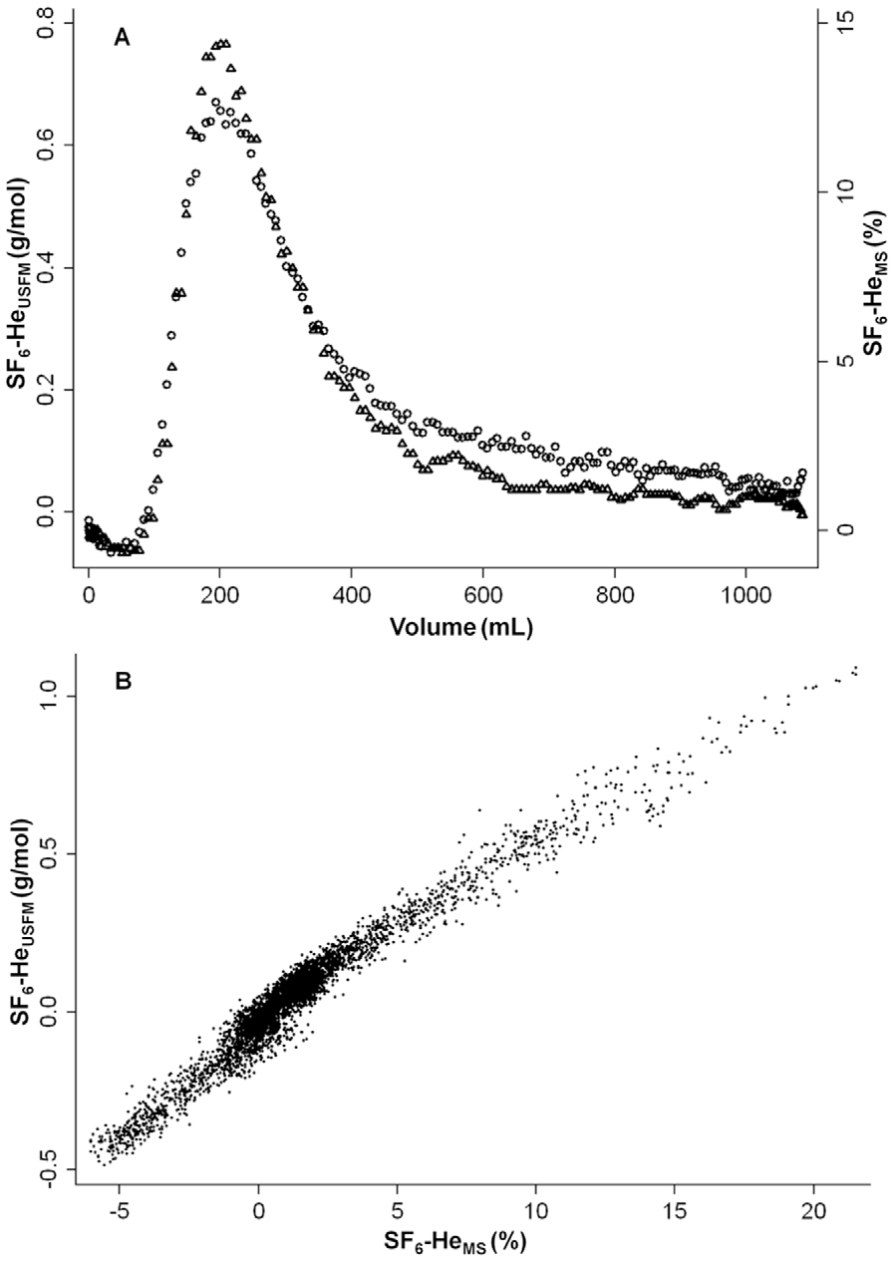
Comparison of sulfur hexafluoride and helium washout curves. Sulfur hexafluoride (SF_6_) and helium (He) washout signals measured using an ultrasonic flowmeter (SF_6_-He_USFM_) and mass spectrometer (SF_6_-He_MS_) derived from a tidal single breath washout (SBW). Figure 5a: Typical SBW signals from one healthy male adult. SF_6_-He_USFM_ (circles) and SF_6_-He_MS_ (triangles) were plotted as expirogram against expired volume. Figure 5b: SF_6_-He_USFM_ was plotted against SF_6_-He_MS_ derived from 18 tidal SBW tests in six subjects. Accounting for clustered data, adjusted linear regression coefficient r^2^ was 0.96 (p<0.001).

Calculation of SF_6_-He_USFM_ and SF_6_-He_MS_ signals was feasible in all DTG-SBW tests. SF_6_-He_USFM_ washout curves were strongly associated with SF_6_-He_MS_ washout curves (figure 5a). Paired data of SF_6_-He_USFM_ and SF_6_-He_MS_ from 18 tests were plotted against each other, the adjusted linear regression coefficient r^2^ was 0.96 (figure 5b). A relative increase in either tracer gas was reliably reflected in the SF_6_-He_USFM_ signal.

To investigate if the shape of the SF_6_-He_USFM_ expirogram was robust against technical factors, we assessed the impact of possible VI caused by the measurement setup and variable dead space, respectively, on the shape of SF_6_-He_USFM_. First, three DTG-SBW were performed using a 500 mL precision syringe at 40 tidal strokes per minute, and a 1000 mL precision syringe at 20 tidal strokes per minute, respectively. DTG-SBW tests (n = 6) in these precision syringes resulted in flat SF_6_-He_USFM_ signals similar to signals of pre-test strokes using air. Second, pre- and post-capillary dead spaces were increased step-wise using 3.5 mL tubes resulting in 17.5 mL additional dead space on either side of the sidestream MM_USFM_ sampling inlet. During each step, one DTG-SBW test was applied in one healthy adult. For each step (n = 10), SF_6_-He_USFM_ expirograms were similar to those recorded using the original setup.

### Reproducibility of the single breath washout method

**Figure 6:**
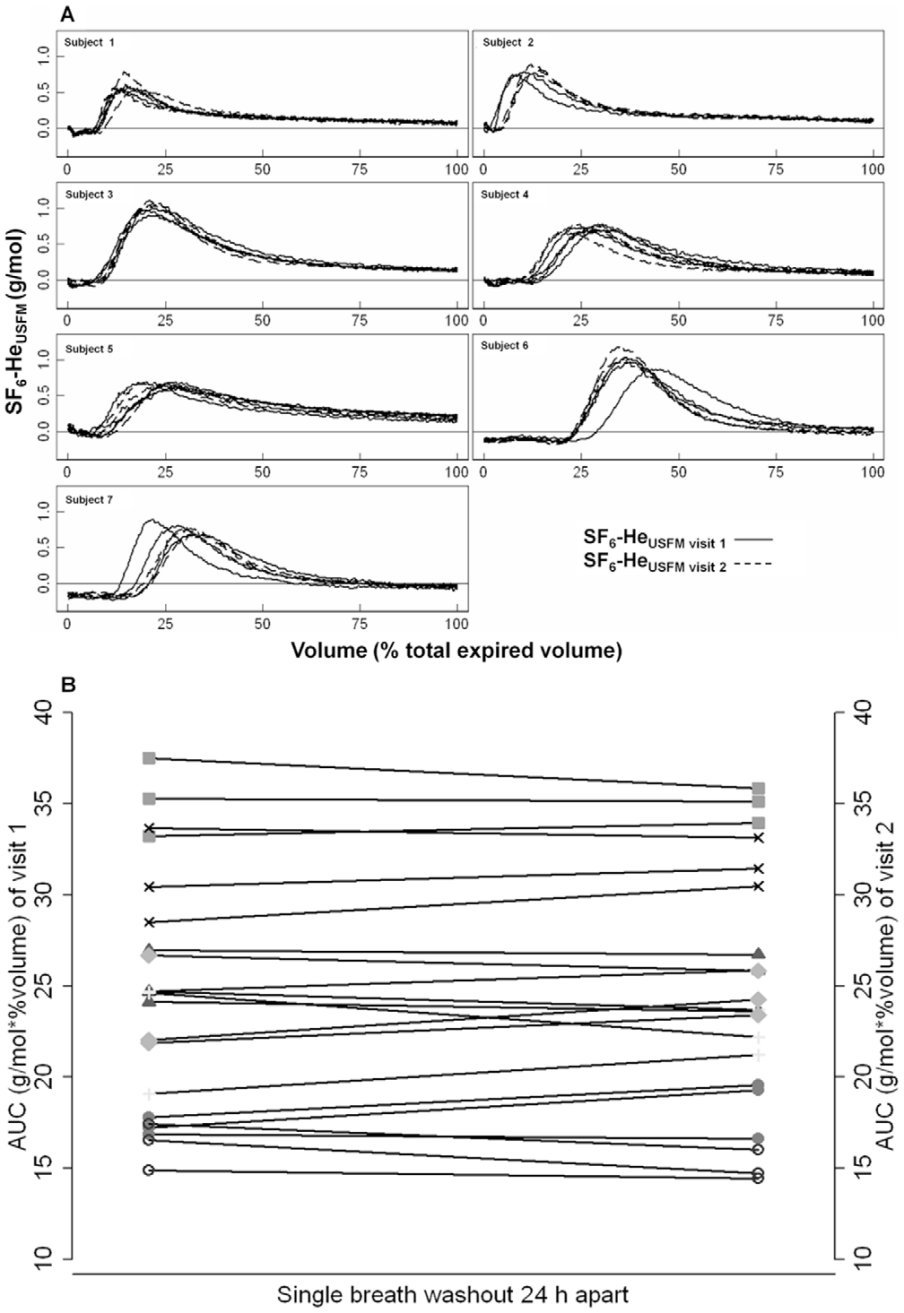
Reproducibility of the single breath washout. Seven healthy adults performed three double tracer gas single breath washout tests (DTG-SBW) 24 hours apart. Ultrasonic flowmeter (USFM) derived sulfur hexafluoride (SF_6_) and helium (He) washout (SF_6_-He_USFM_) signals were plotted as expirogram against percentage of total expired volume. Figure 6a: All SF_6_-He_USFM_ signals of DTG-SBW tests (n = 42) of visit one (black lines) and two (dashed lines) were plotted per subject. Figure 6b: AUC of the SF_6_-He_USFM_ from three DTG-SBW tests were plotted per test occasion and subject with a single symbol for each subject. Intra-individual changes of AUC between visit one and two were tracked via connecting lines.

The shape of SF_6_-He_USFM_ expirograms was repeatable and reproducible (figure 6a). Calculation of AUC was feasible in all tests (n = 42). On day one, mean (SD) AUC was 24.5 (6.7) g/mol*%volume, and on day two, mean (SD) AUC was 24.6 (6.7) g/mol*%volume (figure 6b). Within-test repeatability given as mean (SD) intra-subject CV was 6.8% (3.2%). Between-test reproducibility assessed graphically was good without evidence of systematic bias or significant outliers. The coefficient of repeatability was 2.9 g/mol*%volume corresponding to 11.8% of mean AUC of both visits. Mean (95% CI) difference of AUC between the two test occasions was −0.15 (−0.82 to 0.50) g/mol*%volume.

## Discussion

The USFM accurately measures relative changes in SF_6_ and He washout. DTG-SBW tests are repeatable and reproducible in healthy adults. A tracer gas mixture of similar MM as air is suitable to explore SF_6_ and He washout using an USFM. The shape of the MM expirogram reflects the sequential arrival of SF_6_ and He at airway opening. During a single tidal breath, SF_6_ and He washout patterns are different as hypothesized due to their different physical characteristics and their interaction with normal airway structure. The AUC of DTG-SBW showed high intra-test repeatability and between-test reproducibility in healthy adults.

### Strengths of the single breath washout method

This DTG-SBW method has several strengths with regards to practicability, informative value, and hardware. DTG-SBW tests are easy and quick to perform, requiring only 30 to 40 seconds for a few tidal breaths, and minimal cooperation. Compared to tracer gas SBW tests based on vital capacity maneuvers, classical outcomes, *e.g.* closing volume, are not assessed. However, indices derived from SBW tests near FRC may be even more sensitive for small airway disease [9].

We used low tracer gas concentrations minimizing respective physical interaction and consumption per test [29]. Compared to MBW tests, consumption of SF_6_, a known greenhouse gas, is considerably smaller in SBW tests.

USFM setups are economic and handy, thus probably more suited for clinical routine compared to MS [12]; [16]; [17]; [30]. While the USFM does not allow measurements of single gas concentrations, it accurately measures relative changes of SF_6_ and He at a single spot. Using MS, a delay correction for each gas signal is needed [8]; [22]. With regards to higher signal-to-noise ratio, signal resolution, and lower technical dead space, the USFM technique is probably better qualified than MS to assess gas signals with high fluctuation in *e.g.* infants [15]; [31].

### Accuracy of the single breath washout method

Our findings are in good agreement with previous studies investigating the accuracy of an USFM for MBW using SF_6_ [16]; [17]. *Fuchs et al.* [17] compared the USFM with MS for MBW, and 95% of differences between USFM and MS signals were within a range of <1% of mean SF_6_ concentration. Even not focusing exclusively on end-expiratory tracer gas levels [17], which probably reveals more stable signals, we were able to demonstrate excellent MM_USFM_ signal accuracy (figure 4b). In general it has to be acknowledged that the observed signal differences were well below 1% of the mean MM (figure 4b) and are probably due to inaccuracies of both methods, as no systematic bias was evident.

### Reproducibility of the single breath washout method

The DTG-SBW was highly repeatable and reproducible with low inherent variation in measurements over time. The CV of AUC compares favourably to CV of other tidal breathing or vital capacity techniques. CV of slope of phase III derived from vital capacity N_2_ SBW in children and adults was 13% and 15%, respectively [32]; [33]. In healthy adults, intra-individual change between days in forced expiratory volume in one second (FEV_1_) of 11% is attributed to true clinical change [34]. For DTG-SBW, an intra-individual change in AUC of more than 12% would be unlikely due to measurement noise and thus reflect a true physiological impact with 95% probability [28]. Certainly, more data from DTG-SBW tests in a population of interest are required to estimate significant change.

### Possible mechanisms and physiological relevance

Single tracer gas SBW tests are not specific for small airway disease as these tests do not allow separation between VI due to convective gas transport in large airways and VI due to interaction between diffusion and convection resulting in small airways [20]–[22]. Based on the *Paiva and Engel* lung model [19], the diffusion front for SF_6_ arises more distal than for He. Greater structural asymmetries in lung periphery and the *Peclet* number describing the ratio of convection and diffusion transport of gases contribute to the differing distributions of SF_6_ and He. In healthy subjects these gas transport mechanisms result in a non-linear washout relationship of SF_6_ and He which may be altered in small airway disease most likely due to structural alterations in lung periphery [8]; [9]; [19]; [22]; [35]–[37]. We assume that these mechanisms determined the shape of the DTG-SBW curve in our study. During expiratory phase I representing absolute dead space, a “negative” MM signal reflecting relatively more He than SF_6_ washout was observed (figure 2). Subsequently the “positive” MM signal reflected relatively more SF_6_ than He washout during the bronchial and alveolar phases (figures 2 and 3). These findings are consistent with MS based SBW studies using SF_6_ and He in healthy adults [9]; [21].

### Limitations and open questions

We did not apply DTG-SBW tests in children or diseased subjects. Thus further studies supporting its feasibility are needed. However, tidal breathing lung function tests have been already successfully applied in healthy and diseased young children [6]; [15]; [23]; [30].

As MM_USFM_ depends on humidity and temperature, sidestream sampling was applied to address this issue [17]; [18]; [38]. Compared to mainstream techniques, sidestream sampling introduces signal delay and slightly increases dead space [23]. In our study, system inherent VI and up to 44% increase of technical dead space on either side of the MM sampling tube did not affect the shape of the DTG-SBW curve. This suggests that within these volume limits SF_6_ and He transport mechanisms are not significantly altered by increased dead space, *i.e.* gas bulks of SF_6_ and He may rather flow by convection than diffusion [19]. Further data are necessary to identify potential confounders of the DTG-SBW curve such as breathing pattern, lung volume, and flow [39]; [40]. These issues, however, apply to MBW tests or MS and other USFM setups as well [17].

We propose AUC as first and straightforward index to quantify the shape of the SF_6_ and He washout curve, but suggest that this complex washout relationship would be best explained by appropriate modelling allowing more information on lung physiology to be obtained. Complementary data on airway disease may be gathered comparing DTG-SBW indices with diffusion indices derived from upcoming sophisticated lung imaging techniques, *e.g.* hyperpolarized helium magnetic resonance imaging [41], or additional lung function tests, such as MBW, vital capacity SBW or electrical impedance tomography [42].

### Conclusion

Relative change in SF_6_ and He washout may be viewed as a marker of functional changes in lung periphery, making it potentially sensitive to pathological processes affecting the structure of this ventilation zone. We have developed a fast, reliable, and straightforward USFM based SBW method, which provides valid information on SF_6_ and He washout patterns during tidal breathing in healthy adults. This easy SBW test has potential for widespread use in clinical and research settings.
